# Modern Sociology

**Published:** 1900-02-24

**Authors:** 


### Modern Sociology.

### IS PAUPERISM INCREASING?

By a Correspondent.

In Canon Blackley's evidence before the Royal Commission on the subject of old age pensions, he brought forward statistics to prove that more than 42 per cent. of the deaths of persons over the age of 60 in England were those of paupers. As he put it, nearly every one of us had a corpse chained to his wrist—some one who, from the age of 60 to the time of his death, was maintained by the rates. This seems appalling, and though some part, perhaps a large part, of this pauperism is due to bad habits of those who become paupers, it cannot all be set down to that score. A statement such as this gives a seeming countenance to the outcry of the popular agitator that the increasing wealth of the rich is more than counterbalanced by the increasing poverty of the poor.

Of course, we all know, and the agitator when pressed admits, that this statement is not an accurate one. The poorest now possess many comforts which a century ago were not attainable by those who were comparatively well off. But he says the difference between the conditions of the rich and the poor is now greater than ever. Even that is not a very valuable contribution to sociology, but it impresses the public. But is it true that poverty, as tested by that destitution which is willing to accept poor-law relief with all its drawbacks, is more prevalent than formerly? The answer is, that it entirely depends on the kind of relief. There is no doubt that there is an increasing readiness to accept outdoor relief, and in many quarters there is an increasing willingness, founded on the discussion of this question of old-age pensions, to give outdoor relief. If a person would be a suitable candidate for an old-age pension, if such were to be had, a good-natured board of guardians thinks he is a suitable recipient of outdoor relief. Where this is the attitude of the guardians there is invariably an increase of pauperism; and when we are told that pauperism is increasing, we have a right to ask if it is outdoor or indoor pauperism that is on the upgrade.

As a matter of fact, the two go up together. A man who has received the weekly dole of the parish in his own home, has got so habituated to the idea of charity that he does not shrink from the alternative of the workhouse when some change in his family circumstances makes it impossible to keep up a roof over his head. A family that has been demoralised by permitting their relations to receive outdoor relief loses the sense of pride and independence that forbids them to let the old people go to "the house." There is a general inclination to rely on the parish. To this, as much as to difference of general prosperity, is it due that in some parishes the rate of pauperism, both indoor and outdoor, has been going down for years past, while in others it has gone up.

The difference between districts whose conditions are similar may be shown by reference to different districts in town and country. To take the rural parishes first, we may contrast the parish of Linton, in Cambridgeshire, and Brixworth, in Northamptonshire, the populations of which are not very different. Linton has 12,724 inhabitants, according to the census of 1891, and Brixworth 12,186. Linton shows a record of 110 indoor and 764 outdoor paupers in receipt of relief on January 1st, 1891. Brixworth on that date had 69 indoor and 31 outdoor paupers. Perhaps the model parish in England for lack of pauperism is that of Bradfield, in Berkshire. There, out of a population of 18,017, they have 113 indoor and 29 outdoor paupers, a total of 142, or, in all, one in 126·8 of the population, whereas the proportion of paupers to the total population in Linton was one in 14·5. Twenty years before there had been in Bradfield 1,258 paupers, 999 outdoor and 259 indoor. There is, therefore, a great decrease in both departments, though it is larger in the indoor section. The superior self-dependence of Bradfield, it is evident, is due to no exceptionally good conditions of life for the class that usually falls into pauperism. They would be paupers there if they could manage it comfortably. But they do not fancy uncomfortable pauperism—that of the workhouse.

In London things are much the same, though there we find a much larger proportion of indoor paupers. This is due in great measure to the recent improvements in our poor law infirmaries, which makes people who otherwise neither need nor desire parish help accept treatment in them. A strong contrast is to be found in the unions of Sepney and the Strand. The population of Stepney is more than double that of the other district—57,599 in 1891 against 27,473 in the Strand. Yet the Strand shows a record of 2,304 indoor and 399 outdoor paupers, or a proportion of 1 in 15·8 of the population, whereas Stepney has only 878 indocr and 138 outdoor paupers, a proportion of 1 in 56·6. Yet one would say that the Strand was not such a poor district as Stepney, although, being a central district, it may hold a large semi-vagrant population who readily drift into pauperism. But this will not explain the difference wholly.

Thus it would appear that we cannot draw an exact inference as to the amount of poverty in our midst from the existing amount of pauperism. Guardians who are lax in the administration of relief, especially outdoor relief, encourage pauperism. Does it follow, however, that where the Guardians are strict the poor are left to starve unheeded? It does not. Charitable agencies are so numerous that there is very little chance of any worthy persons, who will own their poverty to the world, being left in destitution. Unhappily, the worthiest of the poor often remain unhelped, because even when they cannot help themselves they will not stoop to beg. But the poor law does nothing for these, and it is the rightful mission of the churches to seek out and help these sufferers. Cases in which poverty springs from undeserved misfortune need not and should not seek pauper relief. And there seems to be no doubt that the employment of the workhouse test tends to encourage thrift by restraining pauperism. Mr. Bland Garland, to whose conscientious administration of the rates Bradfield largely owes its small pauper population, says that there, since outdoor relief has been so sparingly granted, there has been a great increase in the amount deposited in the savings banks and with friendly societies. Keeping this fact in view, we hope that if ever a national old-age pension scheme is instituted it will be based on the sound principle of self-help.

